# Larvicidal and Cytotoxic Potential of Squamocin on the Midgut of *Aedes aegypti* (Diptera: Culicidae)

**DOI:** 10.3390/toxins6041169

**Published:** 2014-03-26

**Authors:** Marilza S. Costa, Jamile F. S. Cossolin, Mônica J. B. Pereira, Antônio E. G. Sant’Ana, Milena D. Lima, José C. Zanuncio, José Eduardo Serrão

**Affiliations:** 1 Laboratory of Cell Ultrastructure, Department of General Biology, Federal University of Viçosa, Viçosa 36570-000, Minas Gerais, Brazil; E-Mails: marilza.costa@ufv.br (M.S.C.); jamile.cossolin@ufv.br (J.F.S.C.); 2 Laboratory of Entomology, Research and Study Center of Agriculture and environment Development, University of Mato Grosso State; MT 358, Km 7, Jardim Aeroporto, Tangará da Serra 78300-000, Mato Grosso, Brazil; E-Mail: monica@unemat.br; 3 Institute of Chemistry and Biotechnology, Federal University of Alagoas, Avenida Lourival Melo Mota, Tabuleiro do Martins, Maceió 57072-970, Alagoas, Brazil; E-Mails: aegs@qui.ufal.br (A.E.G.S.); miladuli@yahoo.com.br (M.D.L.); 4 Departament of Entomology, Federal University of de Viçosa, Viçosa 36570-000, Minas Gerais, Brazil; E-Mail: zanuncio@ufv.br

**Keywords:** acetogenin, Annonaceae, cell death, botanical insecticides, dengue

## Abstract

Acetogenins are secondary metabolites exclusively produced by Annonaceae, which have antitumor, cytotoxic, and pesticide activities. In this study, we evaluated the larvicidal and cytotoxic effect of squamocin from *Annona squamosa* on *Aedes aegypti* (Diptera: Culicidae) midgut. The compound was solubilized in 2% Tween 20 at 10, 20, 50, 80 and 100 ppm. The assay was conducted in a completely randomized design with four replications, each with 20 third-instar larvae. Larval mortality was assessed every hour until total mortality, and the data were subjected to Probit analysis. Cellular damage was evaluated every 30 min in groups comprising five larvae subjected to squamocin at 50 and 100 ppm for 240 min. The total larval mortality occurred after 360 min following application of 50, 80, and 100 ppm squamocin, and 600 min after applying other concentrations with LC_50_ at 6.4 ppm. Both 50 and 100 ppm of squamocin showed cytotoxic activity in the midgut epithelium of *A. aegypti* after 240 min with 50 ppm resulting in midgut cells with light cytoplasm containing small vacuoles, whereas at 100 ppm were found cells with cytoplasm highly vacuolated, damaged apical surface and cell protrusion toward the gut lumen. In conclusion, squamocin has the potential to control *A. aegypti.*

## 1. Introduction

Acetogenins are exclusive secondary metabolites of Annonaceae synthesized via acetic acid-polyketides derived from long-chain fatty acids with 35–39 carbon atoms [1]. These compounds are characterized by a long aliphatic chain with hydroxyl functional groups, and acetyl carbonyl and a terminal γ**-**lactone ring with 1–3 tetrahydrofuran (THF) rings [2].

Squamocin, also called anonin I, is an acetogenin with 37 carbon atoms, α, β-unsaturated γ**-**lactone ring, and adjacent *bis*-tetrahydrofuran (bis-THF) ring [3], and has been reported in *Annona squamosa* and *Annona atemoia* [4].

Acetogenins have cytotoxic, antitumor and pesticide activities [5], and act on cellular models by inhibiting mitochondrial respiration at complex I [6] and NADH: ubiquinone oxidoreductase thus blocking mitochondrial oxidative phosphorylation resulting in apoptosis [7].

These compounds are gut poisons and are particularly effective against chewing insects such as Lepidoptera and *Leptinotarsa decemlineata* (Say) 1824 (Coleoptera: Chrysomelidae) [8].

*Annona squamosa* has been noted to show lethal effect against insects, especially mosquitoes [9]. The crude extracts of *Annona coriacea* (species rich in aromatic acetogenins) have been observed to partially or totally disrupt midgut epithelial cells of *A. aegypti* [10]. However the use of crude extract does not indicate that the Annonaceae acetogenins cause these changes because plants of this family produce other secondary compounds toxic to insects, such as tannins, alkaloids and lectins [11].

The aim of this study was to evaluate the larvicidal and cytotoxic effect of the acetogenin, squamocin, on the larval midgut of the dengue vector *Aedes aegypti*.

## 2. Results and Discussion

After exposure to squamocin at concentrations of 50, 80, and 100 ppm for 360 min, the *A. aegypti* larvae mortality was 100%. Furthermore, all the mosquito larvae died after 190 min of exposure to 10 and 20 ppm of squamocin. The LC_50_ was 6.4 ppm for *A. aegypti* larvae after 190 min.

**Figure 1:**
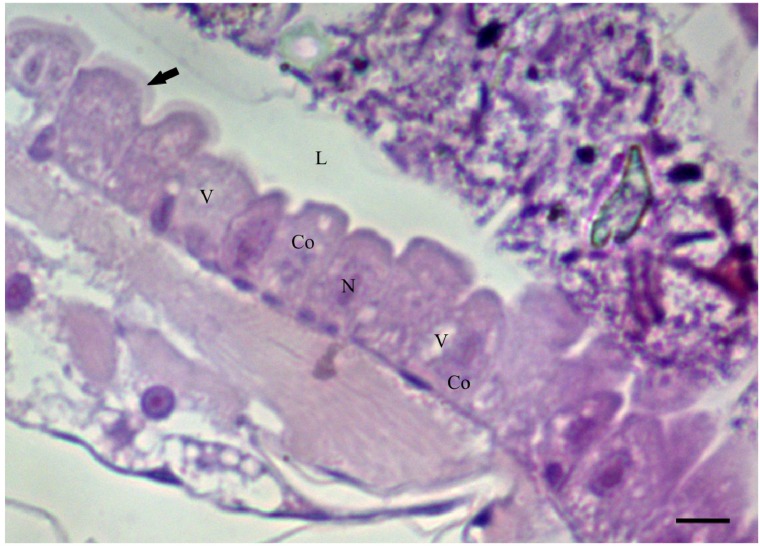
Photomicrograph of the midgut of *Aedes aegypti* third-instar larvae (Diptera: Culicidae) stained with hematoxyline and eosin (HE). Columnar cells (Co), central nucleus (N), some cytoplasm vacuoles (v) and pink brush border (arrow) in the apical surface of the cells in larvae of controls group. L = lumen. Bar: 20 μm.

**Figure 2:**
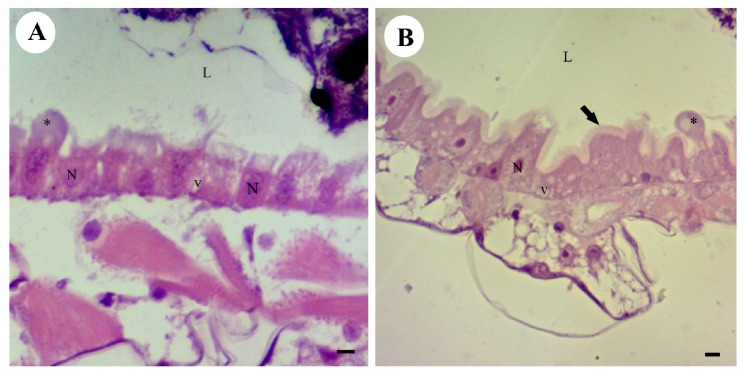
Photomicrograph of the midgut of *Aedes aegypti* third-instar larvae (Diptera: Culicidae) stained with HE. (**A**) Squamocin at 50 ppm for 30 min; (**B**) Squamocin in 50 ppm for 60 min. (L) lumen, (N) nucleus, (v) vacuoles, (arrow) brush border. Bars: 10 μm.

The acetogenin, squamocin, isolated from *A. squamosa*, at concentrations of 50–100 ppm, showed cytotoxic activity with gradual changes in the midgut epithelium of *A. aegypti* larvae from 120 min of exposure. The midgut epithelium exhibited a single layer of columnar cells lining the lumen in the control and insects exposed to squamocin at concentrations of 50 and 100 ppm for 30 and 60 min (Figure 1). The central nucleus of these cells showed decondensed chromatin and an evident nucleolus, the cytoplasm presented some small vacuoles, the apical brush border was found along the entire midgut length, and a peritrophic membrane surrounded the gut content (Figure 2A,B).

**Figure 3:**
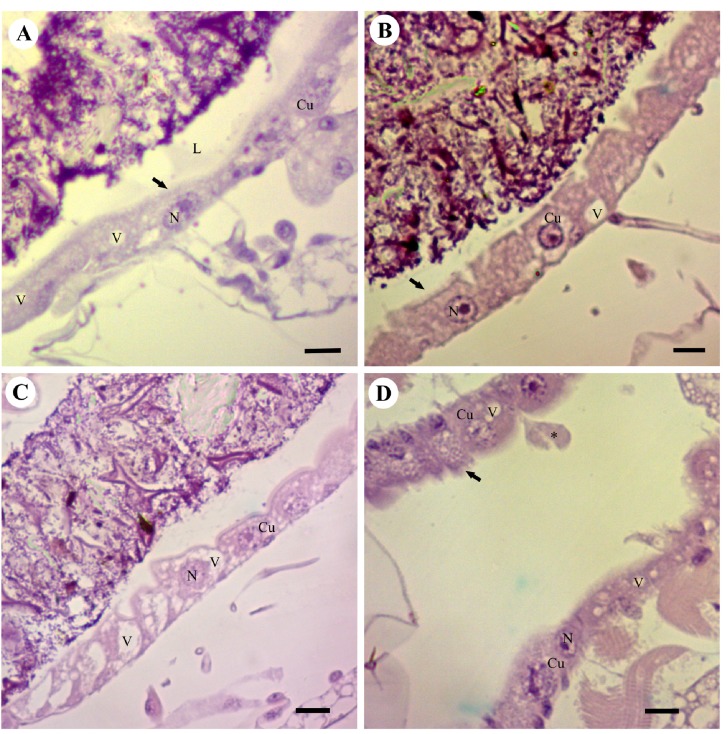
Photomicrograph of the midgut of *Aedes aegypti* third-instar larvae (Diptera: Culicidae) stained with HE. (**A**) Squamocin in 50 ppm for 120 min showing cubic cells (Cu) with central nuclei (N) and vacuolated cytoplasm (v); (**B**) Squamocin in 50 ppm for 240 min showing the cytoplasm with large vacuoles (v) and brush border with apparent damages (arrow) and cytoplasmic projections toward the lumen; (**C**) Squamocin in 100 ppm for 120 and 240 min (**D**) showing large vacuoles (v) and brush border with apparent damage (arrow) and cytoplasmic projections (*) toward the lumen. Bars: 20 μm.

The *A. aegypti* larvae exposed to 50 and 100 ppm of squamocin for 120 and 240 min showed midgut epithelium with cubic digestive cells, which had dilated apical surface, and the cytoplasm containing larger area of vacuolization (Figure 3A,B) than midgut cells in the control insects.

Following exposure to 100 ppm of squamocin for 120 min, the midgut of the *A. aegypti* larvae showed disruption of the peritrophic membrane and damage of the brush border (Figure 3C). After 240 min of exposure to 100 ppm of squamocin, the cytoplasmic vacuoles occupied most of the digestive cells, the brush border was found in different degenerative stages, and the number of cells with apical cytoplasm projections into the midgut lumen was high (Figure 3D).

The larvicidal effect of squamocin at concentration of 10 ppm on *A. aegypti* after 600 min of exposure (6.4 ppm LC_50_) indicated the potential use of this compound as an insecticide, similar to that reported against *Plutella xylostella* [12], *Marcellus eurytides* [13] *Spodoptera littoralis*, *Leptinotarsa decemlineata*, *Myzus persicae* [14], *Oncopeltus fasciatus* [15], and *Spodoptera frugiperda* [16].

The effect of squamocin from *A. squamosa* on *A. aegypti* larvae can be explained by the fact that this compound is a *bis*-THF adjacent acetogenin [2,17], which inhibits NADH: ubiquinone oxidoreductase, preventing electron transport in the mitochondrial complex I [1,18]. The electrons in the mitochondrial compex I prevent the production of ATP and cause the death of the insect by affecting cellular respiration [19,20,21].

Vacuolization and changes in the brush border of the midgut digestive cells of the *A. aegypti* larvae may indicate that these cells were dying, perhaps owing to the toxicity of squamocin, as described in *Culex quinquefasciatus* (Diptera: Culicidae) subjected to synthetic insecticides [22] and *Spodoptera frugiperda* (Lepidoptera: Noctuidae) treated with neem extract [23].

The change in the shape of midgut digestive cells from columnar to cubic in *A. aegypti* was firstly reported to have been caused by acetogenin. This change was not found in the midgut of the third-instar larvae of the *A. aegypti* not exposed to squamocin [24]. However, similar changes were observed in the *A. aegypti* larvae exposed to crude extracts of *A. coriacea* [10] and *Derris* *urucu* (Leguminosae) [25], of which the later has rotenone with action similar to that described for acetogenins [26].

The cytotoxic and larvicidal effects of squamocin on *A. aegypti* larvae might be attributed to the structure of the molecule. It has been reported that the acetogenins with adjacent *bis*-THF rings have high toxicity [3,27], which is favored by the polarity necessary for the cytotoxic activity of acetogenins [28] and might improve the interaction of these compounds with the targets within the cell [29]. The THF ring from squamocin may interact with the phosphate group from the lipid plasma membrane [30,31], causing irreversible damages to the structures and functions of the biological membrane [32], as observed in the midgut digestive cells the of *A. aegypti* larvae.

## 3. Experimental Section

The *A. aegypti* larvae, reared at 28 ± 3 °C, 70% ± 10% relative humidity and 12 h photophase, were obtained from the Laboratory of Entomology, University of Mato Grosso State, campus Tangará da Serra.

The squamocin was acquired from the Research Laboratory of Natural Products Chemistry, Federal University of Alagoas, by using high performance liquid chromatography and nuclear magnetic resonance at 97% of purity and solubilized in 2% Tween 20 in concentrations of 10, 20, 50, 80 and 100 ppm.

The larvicidal bioassay was conducted in a completely randomized design with four replications. Each group comprised 20 *A. aegypti* third-instar larvae, which were kept in a 25 mL glass vial. The larval mortality was assessed every hour from the start of the bioassay until total mortality. The data were submitted to Probit analysis.

The cytotoxic action of squamocin at concentrations of 50 and 100 ppm was evaluated in third-instar larvae subjected to this compound in a 25 mL vial.

Five *A. aegypti* larvae were removed every 30 min during the 240 min of exposure to squamocin and transferred to 4% paraformaldehyde in 0.1 M sodium phosphate buffer pH 7.2 for 24 h. The larvae were dehydrated in a graded ethanol series and embedded in resin JB4. The slices of 4 µm thickness were stained with hematoxyline and eosin (HE) and analyzed under a light microscope.

The controls comprised 20 *A. aegypti* third-instar larvae exposed to 25 mL of vehicle Tween 20.

## 4. Conclusions

The larvicidal activity, disruption of the peritrophic membrane, alteration in the structure of the digestive cells, brush border injury, large areas of cytoplasmic vacuolation and cytoplasmic projections into the midgut lumen, suggested that absorption of squamocin caused cell damages in the midgut followed by the death of *A. aegypti* larvae.
